# *Trans*-spliced Heat Shock Protein 90 Modulates Encystation in *Giardia lamblia*

**DOI:** 10.1371/journal.pntd.0002829

**Published:** 2014-05-01

**Authors:** Rishi Kumar Nageshan, Nainita Roy, Shatakshi Ranade, Utpal Tatu

**Affiliations:** The Department of Biochemistry, Indian Institute of Sciences, Bangalore, India; Johns Hopkins Bloomberg School of Public Health, United States of America

## Abstract

**Background:**

Hsp90 from *Giardia lamblia* is expressed by splicing of two independently transcribed RNA molecules, coded by genes named HspN and HspC located 777 kb apart. The reasons underlying such unique *trans*-splicing based generation of GlHsp90 remain unclear.

**Principle Finding:**

In this study using mass-spectrometry we identify the sequence of the unique, junctional peptide contributed by the 5′ UTR of HspC ORF. This peptide is critical for the catalytic function of Hsp90 as it harbours an essential “Arg” in its sequence. We also show that full length GlHsp90 possesses all the functional hall marks of a canonical Hsp90 including its ability to bind and hydrolyze ATP. Using qRT-PCR as well as western blotting approach we find the reconstructed Hsp90 to be induced in response to heat shock. On the contrary we find GlHsp90 to be down regulated during transition from proliferative trophozoites to environmentally resistant cysts. This down regulation of GlHsp90 appears to be mechanistically linked to the encystation process as we find pharmacological inhibition of GlHsp90 function to specifically induce encystation.

**Significance:**

Our results implicate the *trans*-spliced GlHsp90 from *Giardia lamblia* to regulate an essential stage transition in the life cycle of this important human parasite.

## Author Summary

*Giardia lamblia* is one of the most common causes of diarrhoea across the globe. The disease can result in fatalities especially in small children. The parasite is transmitted by contaminated food through faeco-oral route due to unhygienic habits. The parasite exhibits two stages during its lifecycle; namely cysts and trophozoites. Due to their environmentally resistant hardy nature cysts are transmitted through contaminated food into the human body. Upon entry into the human body they convert into active trophozoites and cause pathogenesis of the disease. In the course of infection within the host, some of the trophozoites convert back into cysts and are released in the environment through the faeces. The mechanisms and signals that convert the parasite from trophozoites to cysts are not yet known. Our study, for the first time, implicates heat shock protein 90 of the parasite in the conversion of trophozoites into cysts in the intestine of the infected human body. Hsp90 is famous for its ability to sense environmental changes and provide cues for stage-switch in related parasites. In addition to providing a glimpse into molecular mechanisms of stage inter-conversion, our results suggest potential new ways of treating this important human infection.

## Introduction

Heat Shock Protein 90 (Hsp90), is a versatile molecular chaperone, involved in diverse cellular processes. It is an essential and evolutionarily conserved chaperone present in both prokaryotes and eukaryotes. Hsp90 have selective set of proteins to chaperone called as clients, which majorly includes transcription factors and protein kinases. Through its interaction with its clients it modulates cell cycle, signal transduction, differentiation, development and evolution [1], [2]. In recent past many new roles have been attributed to Hsp90; like stabilization of genetic variations and transposon mediated mutagenesis [3]. In protists like *Dictyostelium*, *Leishmania*, *Plasmodium*, *Toxoplasma* and *Trypanosoma* Hsp90 has been shown to play an important role in growth and development [4], [5], [6], [7], [8], [9]. In *Plasmodium*, Hsp90 has been established to modulate transition from ring to trophozoite stage [6]. Similarly, in *Leishmania* Hsp90 inhibition results in stage differentiation [5] whereas in *Trypanosoma cruzi* challenging Hsp90 function by using specific inhibitor abrogates the growth of the parasite *in vitro* [8]. In parallel, Hsp90 in *Candida* has been shown to be involved in morphogenesis. Inhibition of Hsp90 mimics the temperature dependent morphogenesis of yeast forms to filamentous forms of *Candida* [10], [11]. All together, these studies highlight the varying function of Hsp90 in the diverse biological systems. Due to its involvement in the range of biological processes, Hsp90 has been proposed as a drug target and its inhibitors as candidate drugs for Malaria and Trypanosomiasis [12], [13], [14].

Hsp90 has a characteristic domain organization which is; amino terminal domain, catalytical middle domain and a dimerizing carboxy terminal domain. The amino terminal domain possesses a binding pocket for ATP/Geldanamycin, it also binds to some co-chaperones. The middle domain houses the catalytic residue ‘Arg’ and also some reports suggest that clients bind through this region [15]. Bridging the amino terminal and middle domain is a variable charged linker; truncation studies report its importance in co-chaperone binding and client maturation [16], [17], [18]. Carboxy terminal domain is responsible for the dimerization of Hsp90 and interaction with TPR domain containing proteins to form a molecular chaperone complex.

*Giardia* is a minimalistic protozoan which is a common cause of diarrhea worldwide. The infection in the mammalian hosts is established upon ingestion of the environmentally resistant, latent cysts [19], [20]. The ingested cysts upon encountering the harsh acidic conditions of the host stomach undergo excystation to form actively dividing trophozoites. These trophozoites colonize the colon of the intestine where they adhere to the epithelial cells and are thus well nourished in a nutrient rich milieu [21]. Some of these trophozoites undergo encystation upon environmental cues that are only partially understood [22]. The precise molecular triggers and mechanism underlining these transitions remain unclear [23].

Previously we have shown Hsp90 from *G. lamblia* to be represented as two separate genes (HspN and HspC) together accounting for N- and C-terminal halves of a canonical Hsp90. We found that the pre-mRNA's corresponding to HspN and HspC are brought together by a *trans*-splicing mechanism that is assisted by complimentary, positional sequences within the individual pre-mRNAs [24], [25]. Based on the cDNA sequence of the full length Hsp90 we proposed that removal of a part of HspN and addition of a segment adjoining to HspC are required to generate GlHsp90, during the splicing mechanism. The evidence for such a reconstruction mechanism at proteomic level was however missing in this study. In the present study, we provide proteomic evidence for the above mentioned *trans*-splicing mechanism by mass spectrometry based sequencing of the critical junctional peptide from GlHsp90 expressed in trophozoites of *G. lamblia*. In addition, we have biochemically characterized the *trans*-spliced full length product with respect to its ability to bind and hydrolyze ATP. As described above, *Giardia* has two alternative life stages, namely trophozoites and cysts. The mechanism of encystation and the molecular players involved in this transition have not been identified so far. We find Hsp90 to play an important role in stage transition from trophozoite to cyst in *Giardia*. We find decrease in the levels of GlHsp90 from trophozoites to cysts by more than 50%. In agreement, pharmacological inhibition of GlHsp90 appears to promote the formation of cysts. All together, in addition to proteomic and biochemical characterization of *trans*-spliced GlHsp90 our results provide important functional insights into its role in regulating the life cycle stages of *Giardia lamblia*.

## Materials and Methods

### Cultivation of parasites

*G. lamblia* parasites were cultured in TYI – S33 supplemented with 10% Adult Bovine Serum and sub-cultured with 5×10^4^ cells per tube from log phase parasites [24].

### Heat shock

5×10^4^ cells were seeded and harvested at log phase for the experiment. Healthy adherent cells were subjected to 40°C or 37°C, for 30 mins in water bath, followed by 1 hour of recovery at 37°C. Following the recovery cells were harvested by chilling on ice for 20 mins and spun down at 700× g at 4°C. For western blot analysis cells were lysed as described previously and clarified. Total protein in the supernatant was estimated by Bradford method of protein estimation. Equal protein was resolved on SDS PAGE, transferred on to nitrocellulose membrane and probed with HspN specific antibody [24]. For real time PCR harvested cells were washed with chilled PBS and total RNA was extracted by TRI-reagent as described by manufacturer's protocol.

### Ingel digestion, mass spectrometry and database searching

A narrow slice corresponding to a GlHsp90 band was cut from the stained SDS-PAGE gel and further sliced into smaller gel plugs. Each samples were processed and analysed by automated nanoflow LC-MS/MS as described previously [26]. The spectra were acquired on a Q-STAR Elite mass spectrometer equipped with Applied Biosystems NanoSpray II ion source. The data was acquired in a data dependent mode, one MS spectrum followed by 3 MS/MS spectra. Data analysis was performed in Analyst QS 2.0 software. For identification of proteins the processed data was searched against *Giardia* database (www.giardiadb.org) [27] using the ProteinPilot 2.0 with precursor and fragment mass tolerances of 0.15 Da, cysteine carbamidomethylation as fixed modification and methionine oxidation, lysine acetylation, glutamine and asparagine deamidation as variable modifications. The resulting MS/MS based peptide identifications were manually verified. The mass spectrometry proteomics data have been deposited to the ProteomeXchange Consortium (http://www.proteomexchange.org) via the PRIDE partner repository [28] with the dataset identifier PXD000795.

### RNA isolation and real time RT-PCR

Total RNA was isolated using TRIZOL (Bioline) as per the manufacturer's protocol. The concentration of RNA was estimated at A_260_ and the purity checked at A_260/280_. Quality and integrity of RNA was assessed on 1% formaldehyde-agarose gel. RNA (2–5 µg) was treated with DNAse I, and then reverse transcribed, using OligodT primers. Prior to quantitative real time PCR, semi-quantitative PCR was performed. The sequence of the primers used for amplification were 5′CGCCCTTCGACATGTGGGAC′3 (forward) and 5′CCGCAGCACGACGCCGC′3 (reverse) for HspN, 5′CGCCCTTCGACATGTGGGAC′3 (forward) and 5′GAACGTGAGCCAGTCGGGAAC′3 (reverse) for Full length GlHsp90, 5′-CCCTATAACTAACACGCAGG-3′ (forward) and 5′-CGATGCGATTCTTCTGGAGC-3 (reverse) for HspC and 5′-GCCCGAGGAGATCCCATGG-3′ (forward) and 5′-CTTGCAGCCGCCGGTGATATG-3′ (reverse) for GAPDH. Each primer concentration and the respective annealing temperatures were standardized. Real-Time PCR reactions were performed in a 25 µl mixture containing 1 µl of cDNA (diluted 1∶2) 1× SYBR Green buffer (Bioline), 400 nM primers, 4 mM MgCl2, 0.2 mM dNTPs mix and 0.025 Unit Taq thermostable DNA polymerase. The annealing temperatures used for N-terminus, full-length, C-terminus GlHsp90 and GAPDH amplifications were 57°C, 61°C, 56°C and 60°C respectively. Real-Time quantitations were performed using the BIO-RAD iCycler iQ5 system. Appropriate no-RT and non-template controls were included in each 96-well PCR reaction (in all experiments n = 3, error bars represents S.D).

### Cloning and purification

Primers used for the full length Hsp90 cloning; sense 5′ – CCCCCGGATCCATGCCCGCTGAAGTCTTCGAGTTC -3′ and antisense 5′-CCCCCGAATTCTCAGTCAACTTCGTCAACGTCCTC-3′ were used. For confirmation of gDNA contamination HspN internal sense primer 5′- GTGTfCGCACTTCCGTGTCG -3′ was used in combination of antisense HspN primer used for qRT-PCR. The amplicons were ligated to pRSET series vector with N terminus 6× His-tag. The positive clone was confirmed by restriction digestion and PCR using respective specific primers. The positive clone was transformed to BL21. PlysS E. coli strain, induced by IPTG and purified using Nickel NTA agarose beads as described in manufacturer's instructions.

### ATP binding studies and ATPase activity

Fluorescence measurements were carried out in a Perkin Elmer fluorescence spectrophotometer using 25 µg of purified HspN in 20 mM Tris (pH 7.4) and 1 mM EDTA. The sample was excited with a wavelength of 280 nm and the emission was scanned between 300–400 nm, at 120 nm/min. The filter width was adjusted to 3.5 and 4.5 nm for excitation and emission respectively. Fluorescence emission at 340 nm was taken for all the calculations. The concentrations of ATP used were varied from 50 µM to 100 µM. The data points were fitted with a curve by a non-linear regression analysis using a single site specific binding to get dissociation constant (K_d_) [12].

ATPase assay was carried out in 40 mM HEPES-KOH, 5 mM MgCl2, pH 7.5. The final concentration of purified HspN used was 0.125 µg and the concentration of ATP was varied from 150 to 5000 µM in a final reaction volume of 10 µl. The reaction was incubated at 37°C for 1 hour and then quenched by adding 1 µL of 0.5 M EDTA. Thin layer chromatography was carried out on polyethyleneimine-cellulose F (Merck) sheets, using mobile phase 0.5 M LiCl, 0.5 mM EDTA and 2N formic acid. The polyethyleneimine-cellulose sheets were dried and analyzed by phosphor imaging. The spots corresponding to phosphate and ATP were quantitated using Image Quant software (Fujifilm). To determine the Hsp90-specific ATPase activity, 150 µM of 17-AAG was used. All values are averages of three separate measurements [12].

### Encystation

Two step encystation protocol was followed [29]. Concentration of Ox bile (Hi-media) was standardized for the optimum number of viable cysts formation in vitro. Log phase grown adherent cells in growth medium was harvested. Number of viable cells were counted using trypan blue in haemocytometer. 5×10^3^ live trophozoites/mL were seeded in Pre-encystation medium. Pre-encystation medium differs from growth medium in the bile and pH. Pre-encystation is completely devoid of bile and pH was set at 7.2. Faetal bovine serum was used in this condition. Cells were incubated at 37°C for 3 days (till confluency). At the end of 3 days, motile cells were removed along with the medium and replaced with pre-warmed encystation medium. Encystation medium contain 10 time excess bile in comparison to growth medium with pH of 7.8. Adult bovine serum was maintained in the encystation medium. In addition, Lactic acid was used in the medium. The cells were maintained in this condition for 2 days. At the end of two days cysts formed was counted by haemocytometer and harvested [29], [30] (in all experiments n = 3, error bars represents S.D).

### Harvesting cysts

Cysts are enriched by water lysis of contaminating trophozoites. Cysts were harvested in sterile conditions, tubes were chilled on ice for 20 mins, and cells were pelleted down at 150× g for 10 mins. The cell pellet obtained was re-suspended in sterile chilled water and incubated in 4°C on end on rotor for 20 mins. After the incubation the cysts were pelleted at 150× g for 10 mins and water lysis was carried out repeatedly for 3 times. The cysts obtained were allowed to stand in 4°C over night. The cysts were pelleted down and viability was counted using trypan blue. The purified cysts were either harvested in trizol or in Tris 20 mM with 1% Triton ×100 buffer for total RNA or protein extraction.

### Antisense

Hsp90 antisense was cloned in “Oct-vector” described previously [31]. The insert was amplified using sense primer 5′- ATGCCCGCTGAAGTCTTCGAGTTC -3′ and antisense primer 5′- TCAGTCAACTTCGTCAACGTCCTC -3′ from total cDNA of the Trophozoites. The constructs were prepared in oct-vector by replacing PPAc Antisense using XhoI and ApaI. The overhangs were blunted using Phusion enzyme. The inserts were ligated (blunt end) and transformed to *E. coli* DH5α and screened for negative orientation clones. The orientation was confirmed by restriction digest pattern using BamHI.

## Results

### Junctional peptide of full length Hsp90 is contributed by non-coding strand of a hypothetical ORF

**Figure 1:**
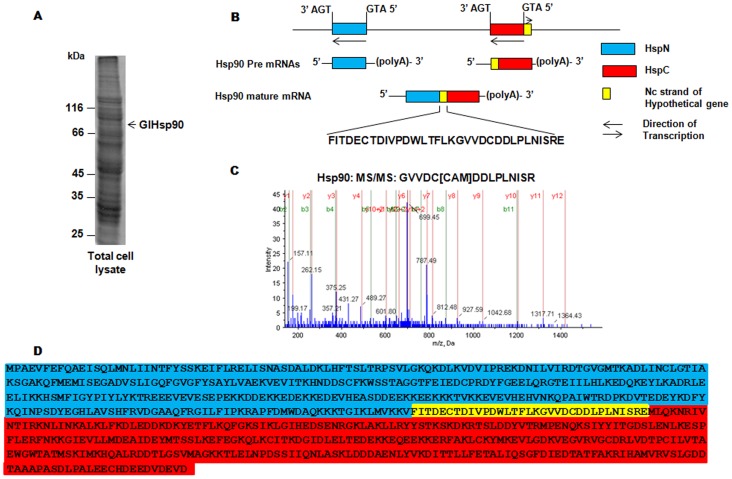
GlHsp90 junctional peptide is contributed by the 5′ UTR of HspC gene. ***A***, Total protein profile of *G. lamblia* trophozoite. The band corresponding to Hsp90 was analyzed by mass spectrometry. ***B*** and ***D***, Representation of the *trans*-spliced full-length mature Hsp90 mRNA and its product respectively. The region highlighted in yellow represents the signature peptide for *trans*-splicing reaction unique to full length Hsp90 which is contributed from 5′ UTR of HspC ORF and also covers the non-coding strand of the hypothetical ORF (GL50803_31962). ***C***, MS/MS spectrum of the signature peptide (a part of junctional peptide, GVVDCDDLPLNISRE).

Based on the cDNA sequence of full length Hsp90 mRNA we have previously proposed that a *trans*-splicing event results in generation of a full length Hsp90 mRNA from two independently expressed HspN (Gene ID, GL50803_98054) and HspC (Gene ID, GL50803_13864) pre-mRNAs. Such a *trans*-splicing event results in removal of a segment of predicted HspN ORF and addition of a junctional peptide from the 5′ untranslated region of predicted HspC ORF. This 5′ region happens to be a non-coding strand of a predicted hypothetical gene in GiardiaDB (Gene ID, GL50803_31692). While we did provide proteomic evidence for the presence of such a full length protein, the precise amino acid sequence of the unique, junctional peptide remained to be shown at the protein level. Towards this, we prepared total cell extracts from *G. lamblia* trophozoites and fractionated the protein mixture on SDS-PAGE (Figure 1A). The position of the full length GlHsp90 protein was known to us from our previous analysis [24]. We thereby performed in-gel trypsin digestion of the GlHsp90 band as described under “materials and methods”. Since the *Giardia*DB does not contain the full-length GlHsp90 sequence, we manually included the protein sequence in our search list by translating the cDNA sequence obtained previously [24]. As expected and shown previously, mass-spectrometric analysis of the tryptic digest revealed the presence of peptides from both the predicted HspN and HspC ORFs (Figure 1C) (other proteins identified from the sample are tabulated in Table S1). In addition, the analysis also revealed the junctional peptide (yellow shaded region on the sequence of Figure 1D) sequence corresponding to the 5′ UTR of HspC ORF, which is also a part of non-coding strand of the predicted hypothetical ORF, GL50803_31692. The above analysis confirms the contribution of 3 independent genes, namely 1) HspN, 2) HspC and 3) non-coding strand of the hypothetical gene in the generation of the full length GlHsp90, the latter two i.e., HspC and non-coding strand of hypothetical gene being expressed as a common transcript.

### *Trans*-spliced Hsp90 is an active ATPase

**Figure 2:**
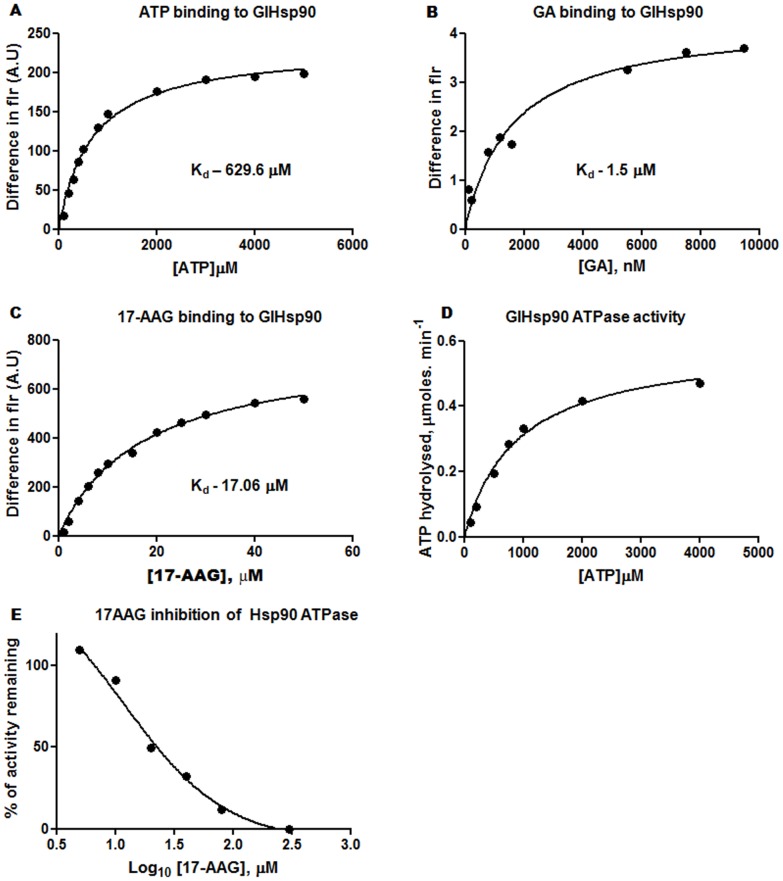
*Trans*-spliced GlHsp90 is an active ATPase. ***A***, ***B***, and ***C***, Binding curve of ATP, GA and 17AAG to full length Hsp90 respectively. The Kd values determined for ATP, GA and 17AAG binding were 629 µM, 1.5 µM and 17 µM respectively. ***D***, Michaelis-Menten plot showing the rate of ATP hydrolysis plotted against ATP concentration. The determined biochemical parameters are tabulated and presented in Table 1. ***E***, 17AAG can inhibit the ATPase activity of Hsp90. EC_50_ value was obtained upon plotting % activity remaining against the Log concentration of 17AAG. EC_50_ value was found to be 11.96 µM.

Hsp90 is an ATP dependent molecular chaperone, which requires binding and hydrolysis of ATP for maturation of the client proteins [32]. According to the annotated sequence of HspN and HspC in GiardiaDB, HspN harbors all the residues required to form the nucleotide binding domain of canonical Hsp90. The catalytic “Arginine” however is absent in either of the predicted ORFs. Only the reconstructed full length Hsp90 generated by *trans*-splicing would have all the features required for binding as well as hydrolysis of ATP. To ascertain whether the *trans*-spliced GlHsp90 is catalytically active and possesses all the features of canonical Hsp90, we cloned, purified and biochemically characterized full length GlHsp90. To analyze whether the *trans*-spliced product could bind and subsequently hydrolyze ATP, we have first determined the binding strength of ATP to Hsp90 *in vitro* by monitoring the decrease in the intrinsic fluorescence of protein upon binding to ligand as described under “materials and methods” In brief, increasing concentration of ATP was incubated with a constant amount of protein and the intrinsic fluorescence was monitored. The saturation curve was obtained when difference in intrinsic fluorescence was plotted against molar concentration of the ligand which was further analyzed by Graphpad Prism 4 software using non-linear regression analysis. The observed K_d_ value for ATP binding was 629.6 µM (Figure 2A) which was in the same range as that of other known Hsp90s (Table 1). Similarly, we have determined K_d_ value for GA and its analog, 17-*N*-allylamino-17-demethoxygeldanamycin (17AAG), and found it to be 1.5 µM and 17.06 µM respectively (Figure 2B and 2C). The binding data confirmed the ability of the *trans*-spliced Hsp90 to bind to its cognate ligand ATP as well its inhibitor GA with high affinity.

**Table 1:** Comparative table, representing kinetic parameters of Hsp90 from diverse biological systems.

Organism	ATP				GA (K_d_ µM)	17-AAG (K_d_ µM)
	K_m_ (µM)	K_cat_ (min^−1^)	K_cat_/K_m_ (min^−1^. µM^−1^)	K_d_ (µM)		
*G. lamblia*	894	4×10^−2^	4.4×10^−5^	629	1.5	17.06
*P. falciparum* *	611	9.9×10^−2^	16.2×10^−5^	168	1.05	-
*H. sapiens* *	324	1.5×10^−2^	4.6×10^−5^	240	4.4	-
*S. cerevisiae* *	511	8×10^−2^	15.6×10^−5^	132	1.2	-

* - adopted from Pallavi *et al.*, 2010 [12].

The binding of Hsp90 to ATP is followed by its hydrolysis which drives the chaperone cycle towards completion and helps in the maturation/stabilization of client proteins [33]. The junctional peptide, that we identified (Figure 1) harbors the key residue which confers Hsp90 its ATPase activity. To assess the biochemical parameters of Hsp90 ATPase activity we have incubated the purified Hsp90 with increasing concentrations of ATP and γP^32^ ATP as tracer and analyzed the rate of hydrolysis by TLC based method as described previously [12]. The data was analyzed in Graphpad Prism 4 using non-linear regression analysis for Michaelis-Menten equation (Figure 2D). The results have been tabulated (Table 1), and as is clear that the *trans*-spliced Hsp90 is an active ATPase, with a catalytic efficiency of 4.4×10^−5^ min^−1^ µM^−1^ and K_cat_ of 4×10^−2^ min^−1^ which was found to be similar to human Hsp90.

### GlHsp90 is up-regulated in response to heat shock

**Figure 3:**
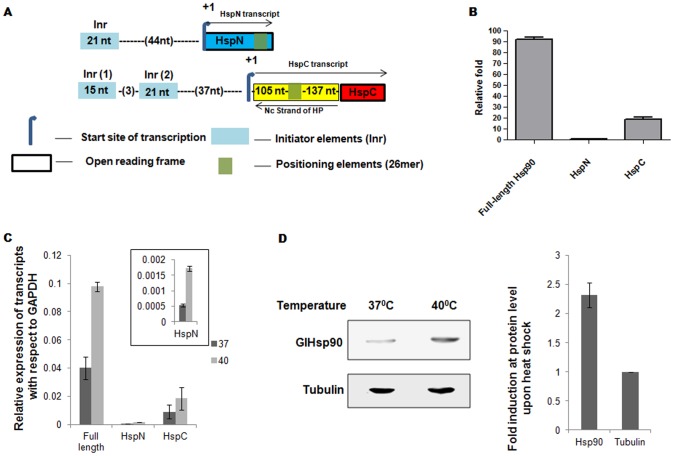
GlHsp90 is induced upon heat shock. ***A***, HspN and HspC promoter elements in blue box (Genbank accession number for HspN and HspC ORFs are AB561868.1 and AB561869.1 respectively), marked in yellow represents the 5′ UTR of HspC, the arrow along HspC ORF is the direction of transcription for HspC ORF. The opposite arrow represents the direction of transcription of the hypothetical ORF (GL50803_31692) which spans the 5′ UTR sequence of HspC in the opposite strand. ***B***, Real-time PCR analysis to study transcriptional expression of full-length Hsp90 and precursor RNAs using specific primers show abundance of full length Hsp90. ***C***, Graph showing the relative levels of induction of full-length, HspN and HspC transcripts from *Giardia* trophozoites as response to heat stress. Inset shows the relative increase in the levels of HspN upon heat shock. Both the precursors and the product were observed to be induced about 2 times at the transcript level. ***D***, Equal proteins from heat shocked and control cells were resolved and probed with anti-Hsp90 antibody. The upper panel represents the signal corresponding to full length Hsp90 whereas lower panel shows the tubulin control blot. The bar graph represents the quantification of the corresponding blot in which GlHsp90 shows 2.3 fold increase (normalized to Tubulin control) as compared to control cells.

Since full length GlHsp90 is generated from two independently expressed transcripts (HspN and HspC), it was important to examine the relative levels of full length and its precursor transcript. Analysis of the upstream sequences of HspN and HspC showed initiation elements (Inr) typically present at −40 to −60 regions of genes in early branching eukaryotes like *G. lamblia* and *Trichomonas vaginalis* [34]. These Inr elements are highly similar to the tubulin and GAPDH genes which have been studied very well in *Giardia* [35], [36] To identify a putative Inr element of Hsp90 gene components we aligned the upstream elements of tubulin with the upstream sequences of Hsp90 gene components. On comparison, it was observed that HspC Inr elements are more similar to tubulin Inr than HspN [35]. In addition to a canonical Inr element of 21 nts found in both HspN and HspC, we found a unique, 15 nts Inr element located at position −61 specifically in HspC ORF. (Figure 3A). The differences in the promoter elements upstream of HspN and HspC ORFs described above suggested differential transcriptional activities of these ORFs.

In order to compare the levels of transcripts of full-length GlHsp90 with those of individual HspN and HspC ORFs, we conducted quantitative real time PCR. Towards this, we have isolated total RNA from *Giardia* trophozoites as described in “materials and methods”. The primers for qRT-PCR were designed against regions which are “specific” to the full-length, HspN, HspC or GAPDH. The specificity of qPCR products was confirmed by melting curve analysis and/or gel-based post-PCR analysis. The genomic DNA contamination was ruled out with PCR for HspN from cDNA prepared with and without RT as control (Figure S1 in Text S1). As can be seen from Figure 3B, the levels GlHsp90 transcript was found to be 90 folds higher than HspN and 5 fold higher than HspC precursors.

To investigate the effect of heat stress on the expression of *trans*-spliced Hsp90, we seeded cells from the stationary phase and subjected them to heat shock at 40°C for 30 mins and maintained the control cells at 37°C as described under “materials and methods”. Following heat shock, cells were recovered at 37°C for 1 hour and harvested as described previously. We quantified the degree of modulation of Hsp90 and its gene components at the transcript level under heat stress using real Time PCR. Towards this we isolated RNA and prepared total cDNA. We used equal amounts of cDNA from heat shock treated and control cells and compared the Ct values for full-length, HspN and HspC. The amplification of GAPDH transcript was used as a control which did not get affected upon heat stress. We analysed our data by comparing Δ Ct and Δ (Δ Ct) values for full-length, HspN and HspC. The relative fold induction of all three transcripts has been represented in the graph as shown in Figure 3D. Upon heat shock, full-length Hsp90, HspN and HspC transcripts were found to be induced at 2.4, 3.2 and 2 folds respectively. To examine if the observed up-regulation of the Hsp90 transcript upon heat shock also hold true at the protein level, we probed cell lysates of trophozoites with and without heat shock with α-Hsp90 antibody. Equal amount of protein was loaded and the fold up-regulation was determined by western blot analysis. It was observed that the increase in Hsp90 was about 2.3 fold at the protein level (Figure 3D, right panel). The above data suggest that despite the split nature of Hsp90 gene, *Giardia* has retained its ability of Hsp90 heat induction through an intricate co-ordination of transcription and *trans*-splicing events. However, the factors contributing towards mRNA and protein stability may also play a role in observed increase in the levels of Hsp90 at transcript and protein level respectively.

### Hsp90 modulates encystation

*Giardia* has a biphasic life cycle with a proliferative trophozoite stage and a latent cyst stage [19]. The molecular machinery and mechanisms involved in the above stage transition remain unclear. As Hsp90 is known to be involved in the ability of eukaryotic cells to sense and respond to environmental changes [10], [37], we examined its potential involvement in *Giardia* stage transition.

**Figure 4:**
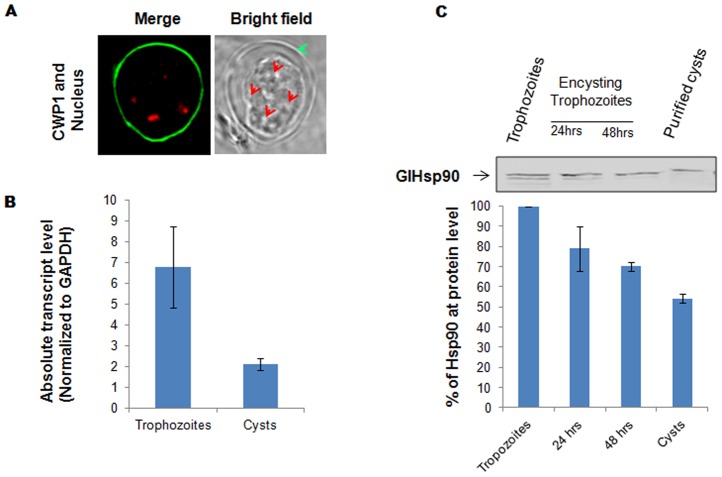
GlHsp90 is down-regulated during encystation. ***A***, The mature cysts show characteristic cyst marker, CWP1 in upper panel with four nuclei which are used for qRT-PCR and western blot analysis. ***B***, Quantitative RT-PCR results show that the level of Hsp90 and its gene components get modulated in cyst stage in comparison with trophozoites. The values are normalized to internal standard, GAPDH, and represented. ***C***, Western blot analysis of Hsp90 during the course of encystation show steady decrease in Hsp90 levels. Quantification of the western blot show that cysts produce 50% less Hsp90 than the active trophozoites, at the beginning of the encystation.

Towards this, we first established encystation process in the axenic culture of *Giardia* trophozoites as described under “materials and methods”. According to previous reports we were able to obtain *Giardia* cysts by modulating pH and bile concentration of the medium [30]. The presence of mature water resistant cysts was confirmed by IFA using α-Cysts Wall Protein 1 (CWP1) antibody, a cyst specific protein, and counter stained for nucleus using propidium iodide [38], [39]. As shown in Figure 4A, in the upper panel, CWP1 shows a characteristic ring like pattern around the cyst and four nuclei are seen in our IFA analysis.

We examined the levels of GlHsp90 both at transcript and protein levels. qRT-PCR was performed as described under “materials and methods” from trophozoites and mature cysts to compare the levels of GlHsp90 transcript. Briefly, equal number of trophozoites and cysts were harvested and RNA was prepared using Trizol as described under “materials and methods”. Equal concentration of RNA corresponding to cyst and trophozoite was used to prepare corresponding cDNAs and subjected to qRT-PCR. The resulting Ct values from trophozoites and cysts reactions were normalised to GAPDH level in respective samples. As can be seen in Figure 4B, we found GlHsp90 to be significantly down-regulated in cysts in comparison to trophozoites. Specifically, GlHsp90 transcript showed 3.5 fold down-regulation in cysts as compared to trophozoites.

To test if the qRT-PCR observation also reflects at GlHsp90 protein level, we have carried out western blot analysis of trophozoites and cysts. We resolved equal amount of protein from encysting trophozoites at different time intervals (0, 24 and 48 hours) [38], until completion of encystation, on SDS-PAGE and transferred onto nitrocellulose membrane. The membrane was probed with GlHsp90 specific antibody and developed appropriately. As shown in Figure 4C, we found gradual decrease in GlHsp90 levels during the course of encystation. The mature cysts showed almost 50% reduction in GlHsp90 protein levels as compared to trophozoites. This result was in agreement with the qRT-PCR data shown above.

To test if the drop in GlHsp90 levels during trophozoites to cysts transition was mechanistically linked to encystation process, we examined the effect of pharmacological inhibition of GlHsp90 on encystation. We used a semisynthetic derivative of Geldanamycin namely 17AAG which was previously shown to inhibit *Giardia* growth with an IC_50_ of 711 nM [24].

**Figure 5:**
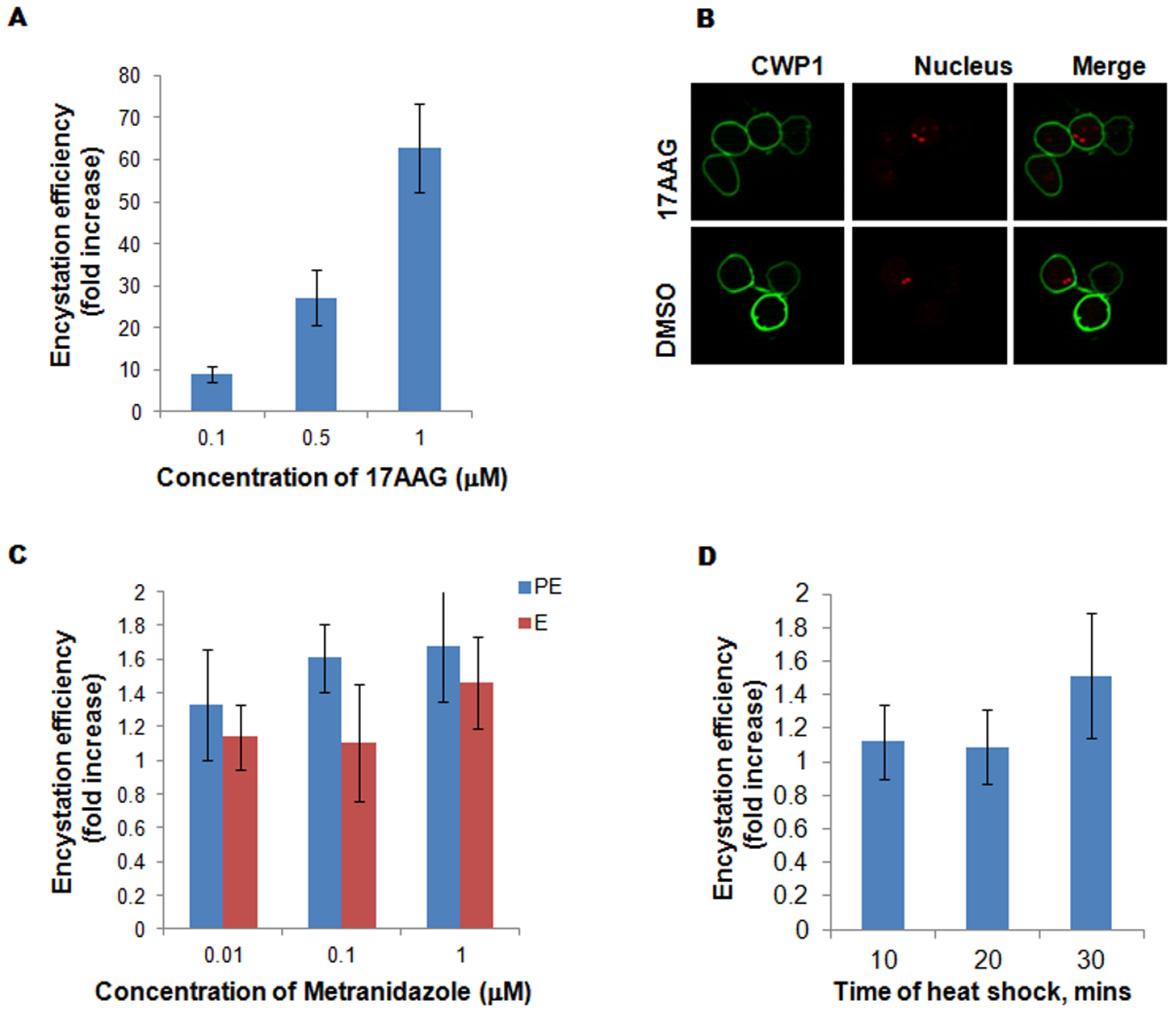
Inhibition of GlHsp90 induces encystation. ***A***, Encystation efficiency of trophozoites as a function of Hsp90 inhibition by 17AAG. Step1 shows an increase in cyst formation upon Hsp90 inhibition at sub-lethal level. Trophozoites treated with varying concentration of 17AAG show transformation to be dose dependent (with 9, 27 and 62 fold cyst formation at 0.1, 0.5 and 1 µM of inhibitor). ***B***, the cysts formed from the treated cultures showed all the characteristic features of a mature cyst as shown in upper panel with specific staining of CWP1 of cyst wall component and 4 nuclei, representing the features of mature cysts. ***C***, Determination of encystation efficiency of cells treated with metranidazole before (blue bar) and during (red bar) encystation do not produce any better cysts than the normal trophozoites. ***D***, Encystation efficiency did not change in response to heat shock. Varying duration of heat shock followed by 90 mins recovered cells encyst similarly as the normal cells maintained at 37°C.

As a pre-requisite we determined the IC_50_ of 17AAG during pre-encystation conditions (Figure S2A in Text S1). In the previously described two step protocol of encystation, we incorporated sub-lethal concentrations of 17AAG (0–1 µM) on the last day of step1 (pre-encystation) and progressed with step 2 (encystation) as described under “materials and methods”. We scored for the number of cysts formed against total number of cells (encystation efficiency) at the end of encystation as described under “materials materials and methods” [40]. As shown in Figure 5A, cells treated with 17AAG on the last day of pre-encystation showed 60 folds higher efficiency as compared to the control cell (DMSO treated). We confirmed that 17AAG induced cysts possess all the characteristic features of a mature cyst by examining expression of CWP1 by IFA. As shown in Figure 5B, 17AAG induced cysts showed characteristic staining for CWP1 antibody. The results indicated that down-regulation of GlHsp90 could be a potential trigger for encystation.

We also attempted genetic approach for down-regulation of GlHsp90 using an antisense approach as explained under “materials and methods”. Upon selection, i.e, scoring for puromycin resistance, live parasites were obtained in vector control; however, no viable parasites were obtained in Hsp90 antisense (data not shown).

We confirmed that the stage transition was indeed a specific result of Hsp90 inhibition and not a general stress response, by carrying out encystation from trophozoites which have undergone other stressful conditions like drug pressure using metranidazole [41], [42], and thermal stress by heat shock [43]. As a preliminary study we determined the IC_50_ value of metranidazole in the pre-encystation condition as represented in Figure S2B in Text S1. Under the similar conditions, encystation was set up and the treatment was carried out both at the last day of step 1 and the number of cysts were counted at the end of encystation. As represented in Figure 5C, the encystation ratio remained similar in all the concentrations of metranidazole used, suggesting that encystation in *Giardia* was not a general response to drug treatment. To understand whether or not thermal shifts had any role in encystation by modulating Hsp90 activity, we carried out encystation of the parasites exposed to heat shock and found that heat shock did not affect formation of cysts in these conditions (Figure 5D). Both the above results indicate that the phenomenon of encystation might not be a general stress response in *Giardia*.

## Discussion

Hsp90 is an essential molecular chaperone in all eukaryotes studied so far. Many regulatory proteins like transcription factors and protein kinases are known to be dependent on Hsp90 for their activity and regulation. Hsp90 has been shown to possess none, one or sometimes multiple *cis*-spliced introns in most organisms studied. However, *G. lamblia* Hsp90 is coded by two ORFs that are spliced in *trans* [24], [25]. The *trans*-splicing phenomenon is different from the classical *trans*-splicing described in *Trypanosomes*, wherein a leader sequence is spliced to an exon. In *Giardia*, messages from two independent ORFs get spliced in *trans* to form full-length Hsp90 mRNA. The resulting mature mRNA coding for full-length Hsp90 protein has a unique junctional sequence encoding 33 amino acids which is not present in either HspN or HspC ORFs. The junctional sequence so formed is contributed by the 5′ UTR of the HspC transcript, which is also the sequence covering the non-coding strand of a predicted Hypothetical ORF. This junctional peptide houses the catalytic argenine which is known to be essential for the ATPase activity of Hsp90 [44]. Previously we have predicted that the full length Hsp90 possess all the essential features in its primary structure for its function; however, no functional role was ascribed to the *trans*-spliced GlHsp90.

While the essential features of the *trans*-splicing reaction were described previously, the formal evidence for the junctional peptide sequence was not provided. Using proteomic approaches, in the present study, we have sequenced the junctional peptide present in between the HspN and the HspC sequence. The presence of the junctional peptide in the parasite is important for the functionality of the *trans*-spliced GlHsp90 as residues in this region are conserved and shown to play an important role in Hsp90 ATPase activity [44].

The Hsp90 transcript formed through *trans*-splicing possesses all the functional signatures for binding and hydrolysis of ATP. ATP hydrolysis is an essential step which fuels the Hsp90 chaperone cycle. As evident from our biochemical assays using purified *Giardia* Hsp90, the *trans*-spliced Hsp90 bound cognate ligand ATP and its inhibitor 17AAG and GA with high efficiency. The binding affinity (K_d_) was found to be 629 µM for ATP and 1.5 µM and 17.06 µM for GA and 17AAG respectively. The bound ATP was further hydrolyzed through the residues from the junctional peptide with catalytical efficiency of 4.4×10^−5^ min^−1^. µM^−1^. The biochemical parameters of GlHsp90 ATP hydrolysis were found to be similar to those of human Hsp90 (4.6×10^−5^ min^−1^. µM^−1^) [12].

*Trans*-splicing of full length Hsp90 mRNA from two independent pre-mRNAs must require co-ordination between transcription of individual ORFs and their subsequent splicing. Bioinformatic analysis of upstream region of HspN and HspC revealed presence of independent promoters with HspC showing the presence of an additional Inr in comparison to HspN. Analysis of the transcripts by real time qRT-PCR confirmed this pattern with HspC transcripts showing 15 folds abundance over HspN. Hsp90 is known to be induced upon various stress conditions [1]. How would the presence of split Hsp90 gene affect its transcriptional upregulation in response to heat shock? In addition to upregulating the individual HspN and HspC transcripts, their *trans*-splicing machinery will also have to cope with such increased levels of these precursor transcripts. Our qRT-PCR and western blot experiments from the heat stressed cells showed significant up-regulation of not only the precursors, HspN and HspC, but also the *trans*-spliced product GlHsp90 at transcript and protein levels. The increased levels of precursors are the substrates of spicelosomal machinery for the formation of full length Hsp90. It is important to note that core spliceosomal components (Table S2) show no significant change upon heat shock, as described in GiardiaDB. However, the spliceosomal machinery was able to cope with increased levels of precursors towards the formation of full length Hsp90, required under stress.

Previous reports from *Leishmania*, *Plasmodium* as well as *Candida* suggest an important link between Hsp90 function and stage transitions in these parasites. For instance, in *Leishmania* it has been shown that Hsp90 regulates stage transition from the promastigote (insect stage) to amastigote (mammalian stage). Invasion of mammalian host by *Giardia* is marked by conversion of the parasite from environmentally resistant, latent cysts to actively growing trophozoites that causes pathogenesis of the disease. The excystation and encystation events in the parasite life cycle have been established *in vitro* through manipulation of pH and bile concentration in the growth medium; however, the molecular mechanism and triggers effecting these transitions have not been fully understood [22], [45]. Analysis of GlHsp90 transcript and protein levels during trophozoite to cyst transition suggest that GlHsp90 may be involved in triggering this transition. Indeed inhibition of GlHsp90 function in actively proliferating trophozoites robustly induced their transformation to cysts. Specifically, 17AAG pre-exposed trophozoites successfully transformed to cyst forms upon encystation cues. Hsp90 induced stage conversion could not be reproduced with other pharmacological agent or environmental perturbation tested by us, suggesting that GlHsp90 may be mechanistically involved in this cellular transformation. Our efforts to genetically turn down GlHsp90 expression to validate this observation were unsuccessful as *Giardia* transfectants expressing antisense constructs of GlHsp90 did not remain viable.

It must be emphasized that the induction of encystation upon treatment with Hsp90 inhibitors is dependent on the timing of its exposure to *Giardia* trophozoites. It was important to expose *Giardia* to Hsp90 inhibitors in the pre-encystation stage to see the robust induction of encystation. Exposure to Hsp90 inhibitors following pre-encystation, during encystation step did not result in any increase in the number of cysts. The observation rules out the toxicity or stress related effects of Hsp90 inhibitors and suggests that perturbation of Hsp90 activity through its inhibitors was priming *Giardia* trophozoites towards encystation. While the downstream events upon Hsp90 inhibition resulting in induction of encystation remain unclear, our study for the first time implicates this important chaperone in regulating stage transition on *Giardia*.

## Supporting Information

Table S1 Proteins identified by Mass-spectrometric analysis of 80 kDa band.
(XLSX)

Table S2 Modulation of spliceosomal components upon heat shock as reported in GiardiaDB.
(XLS)

Text S1 Supporting figures. **Figure S1:** Confirmatory PCR for HspN transcript. The specific amplicon is present only +RT (reverse transcriptase) lane whereas the – RT reaction did not give any amplification, confirming genomic DNA free RNA used for qRT-PCR. **Figure S2:** ***A***, Response curve of trophozoites against 17AAG concentrations. Viable number of trophozoites were counted using trypan blue exclusion method, % viable cells were plotted against Log 17AAG concentration to calculate IC_50_ in the pre-encystation condition. The IC_50_ was determined to be 1.4 µM. Concentration of 17AAG at and below IC_50_ was used to determine encystation efficiency. ***B***, Response curve of trophozoites against Metranidazole concentrations. The IC_50_ growth was determined to be 2.1 µM in the pre-encystation condition.
(DOC)
