# Supplementary material for: Trans-spliced Heat Shock Protein 90 Modulates Encystation in Giardia lamblia
Source: PLoS Negl Trop Dis. 2014 May 1;8(5):e2829. doi: 10.1371/journal.pntd.0002829 (PMC4006730; doi:10.1371/journal.pntd.0002829)
Supplement: Text S1 — Supporting figures. Figure S1: Confirmatory PCR for HspN transcript. The specific amplicon is present only +RT (reverse transcriptase) lane whereas the – RT reaction did not give any amplification, confirming genomic DNA free RNA used for qRT-PCR. Figure S2: A, Response curve of trophozoites against 17AAG concentrations. Viable number of trophozoites were counted using trypan blue exclusion method, % viable cells were plotted against Log 17AAG concentration to calculate IC50 in the pre-encystation condition. The IC50 was determined to be 1.4 µM. Concentration of 17AAG at and below IC50 was used to determine encystation efficiency. B, Response curve of trophozoites against Metranidazole concentrations. The IC50 growth was determined to be 2.1 µM in the pre-encystation condition. (DOC) [file pntd.0002829.s003.doc]

SUPPLEMENTARY FIGURES

FIGURE S1:


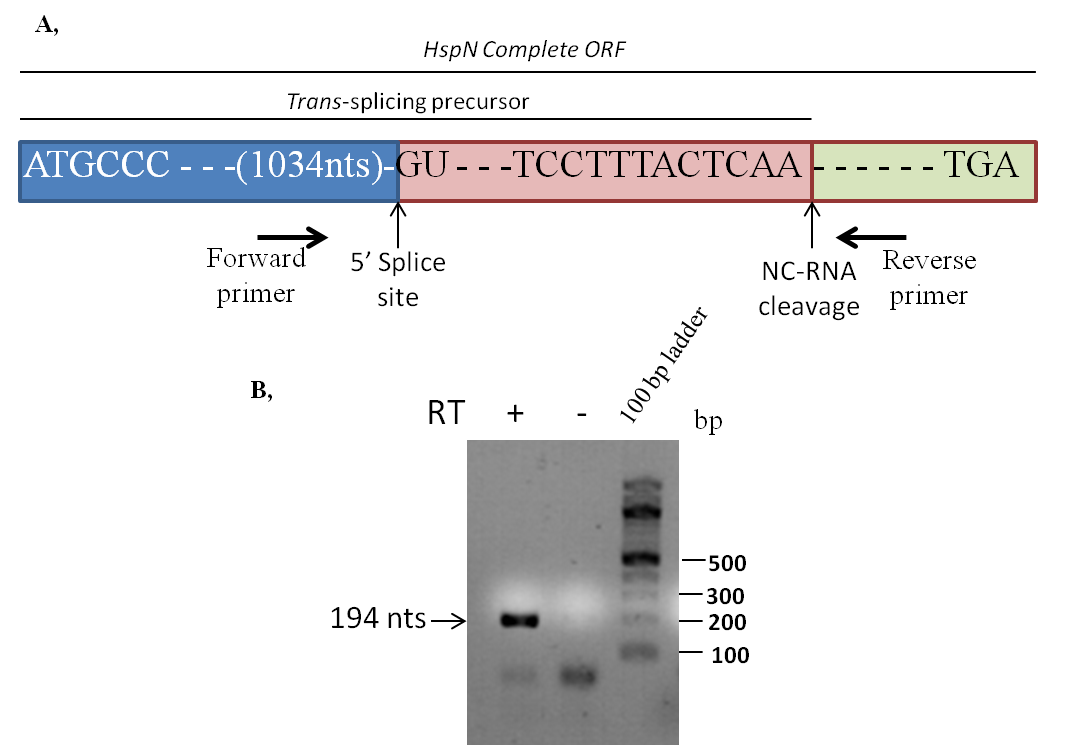


**FIGURE S1:** **Confirmatory PCR for HspN transcript**: The specific amplicon is present only + RT (reverse transcriptase) lane whereas the – RT reaction did not give any amplification, confirming genomic DNA free RNA used for qRT-PCR.

SUPPLEMENTARY FIGURES

FIGURE S2:

A, B,

**
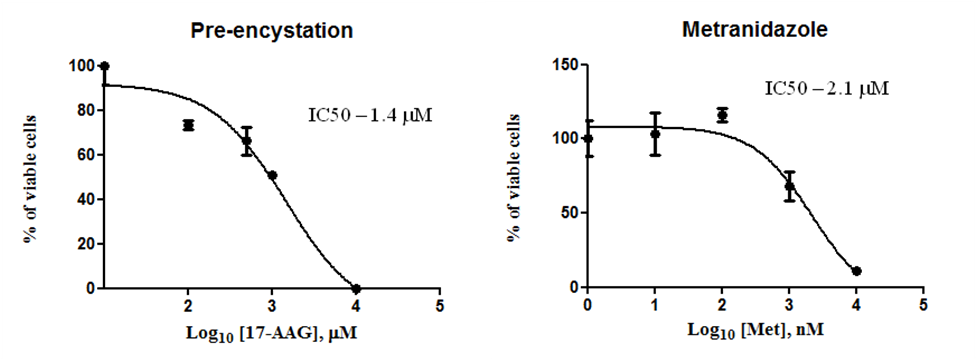
**

**FIGURE S2:** ***A***, Response curve of trophozoites against 17AAG concentrations. Viable number of trophozoites were counted using trypan blue exclusion method, % viable cells were plotted against Log 17AAG concentration to calculate IC_50_ in the pre-encystation condition. The IC_50_ was determined to be 1.4 μM. Concentration of 17AAG at and below IC_50_ was used to determine encystation efficiency. ***B***, Response curve of trophozoites against Metranidazole concentrations. The IC_50_ growth was determined to be 2.1 μM in the pre-encystation condition
